# The Evolving Paradigm of Esophageal and Esophagogastric Junction Adenocarcinoma: Current Insights, Emerging Therapies, and Future Directions

**DOI:** 10.26502/jcsct.5079275

**Published:** 2025-10-27

**Authors:** Regan Laird, Anshu Aggarwal, Devendra K. Agrawal

**Affiliations:** 1 Department of Translational Research, College of Osteopathic Medicine of the Pacific, Western University of Health Sciences, Pomona, CA 91766, USA; 2 Department of Biology and Environmental Sciences, College of Science, Auburn University at Montgomery, Montgomery, AL 36117, USA

**Keywords:** Adenocarcinoma, Barrett’s esophagus, Dysplasia, Esophageal cancer, Esophagogastric junction adenocarcinoma, High-grade dysplasia, Low-grade dysplasia, Metaplasia, Neoadjuvant therapy, Neoplasia, PD-1 antibodies, Tyrosine kinase inhibitors

## Abstract

Adenocarcinoma of the esophagus and esophagogastric junction is a highly aggressive malignancy with a significant mortality risk and poor overall prognosis. The annual incidence of esophageal adenocarcinoma has risen substantially in recent decades and is now recognized as the most common form of esophageal cancer. Early detection of esophageal adenocarcinoma remains challenging due to the frequency of asymptomatic disease progression and ongoing limitations of current screening guidelines. Barrett’s esophagus is the established precursor lesion for esophageal adenocarcinoma. Despite the high mortality rate of esophageal adenocarcinoma, neoplastic progression of Barrett’s esophagus is poorly understood. While the presence of dysplasia can help identify the relatively small subset of patients with Barrett’s esophagus at a higher risk of progression, it is far from a perfect predictor. More research is needed to understand the underlying mechanisms precipitating malignant transformation. Optimal management of esophageal adenocarcinoma requires a coordinated, multidisciplinary approach to tailor risk-stratified screening algorithms and ensure timely intervention. Advancements in treatment protocols, molecularly targeted therapies, and palliative care have improved perioperative outcomes and quality of life. Even still, long-term survival is poor, and recurrence is frequent. Ongoing translational research is essential for reducing disease burden, improving treatment durability, and extending progression-free survival for patients with esophageal adenocarcinoma. This comprehensive review will detail the established guidelines, recent updates, and deficits surrounding the history, diagnosis, staging, treatment, and prognosis of esophageal adenocarcinoma.

## 1. Introduction

Adenocarcinoma of the esophagus and esophagogastric junction (EGJ) is a highly lethal form of cancer, with an overall 5-year survival rate of approximately 20% and a median overall survival of 15 months in the United States [1,2]. The incidence of esophageal adenocarcinoma (EAC) in the United States has increased by more than 20% since the early 2000s, with an annual incidence of 2.8 per 100,000 in 2022 [3]. The high mortality rate of EAC is likely attributable to the high ratio of late-stage diagnoses, with up to 90% of patients receiving an initial diagnosis outside of surveillance programs [4]. In this setting, a clear understanding of the factors contributing to disease progression is key to improving early detection mechanisms. Esophageal cancer typically begins in the mucosa of the esophagus and spreads through deeper tissue layers (i.e., the submucosa, muscle layers, and serosa). Simultaneously, there may be lymphatic or hematogenic progression, the risk for which increases in tandem with the depth of invasion. Adenocarcinoma usually develops in the lower (distal) part of the esophagus at the EGJ [5]. Long-term hyperacidity and uncontrolled chronic gastro-esophageal reflux disease (GERD) due to acid and bile reflux have been linked to the initiation of phenotypic changes in the cells of the distal esophagus. Despite the strong correlation between GERD and the development of EAC, nearly half of patients are asymptomatic and without a prior history of reflux at the time of diagnosis [6,7]. More recent studies have shown that patients with non-erosive GERD have a comparable incidence of EAC to the general population [8]. While individual host factors appear to contribute to the pathogenesis of EAC, their involvement remains undefined.

## 2. Barrett’s Esophagus

### 2.1. History

Barrett’s esophagus (BE) is the precancerous phenomenon preceding EAC, first described by Dr. Norman Barrett in 1950 [9]. BE is caused by intestinal metaplasia of the distal esophagus characterized by a transition from the normal stratified squamous epithelium to an intestinal-type columnar epithelium with goblet cells [10]. The best understood risk for BE is chronic GERD, in which gastric contents reenter the esophagus from the gastric cardia [11]. Over time, this chronic exposure to acidic and noxious agents leads to metaplastic changes to the esophageal epithelium. The resultant cellular metaplasia of BE is a well-understood protective adaptation of the epithelial cells, as columnar cells confer a greater survival advantage within the harsh gastric environment [12]. Due to the accumulation of genetic and epigenetic changes, the metaplastic cells become disorganized and abnormal over time, a condition known as dysplasia. Additional risk factors for BE include older age, male sex, obesity, history of tobacco smoking, hiatal hernia, and Caucasian ethnicity, all of which are also associated with increased risk of EAC [13–15]. Alternatively, *Helicobacter pylori* colonization, specifically strains containing cytotoxin-associated gene A (cagA) is associated with decreased risk of BE and EAC [16]. This is possibly due to the role *H. pylori* plays in the reduction of gastric acid. BE alone is not associated with any symptoms; however, in the setting of chronic reflux, patients typically report symptoms of heartburn, indigestion, dysphagia, chronic cough, sore throat, chest pain, or a persistent bitter/sour taste in the mouth [17,18].

### 2.2. Neoplastic Progression

BE carries an absolute annual risk for neoplastic progression of 0.12–0.5% and a lifetime risk of 3–5% [19,20]. The premalignant morphological changes can be visualized histologically as dysplasia based on key cellular structural and cytological abnormalities. The risk for progression increases in proportion to the degree of dysplasia. In low-grade dysplasia (LGD), the epithelial cells become more elongated and crowded with abnormal orientation, disorganization, and proliferation and contain enlarged, hyperchromatic and irregular nuclei primarily in the basal layers of the epithelium. High-grade dysplasia (HGD) is the most-advanced pre-cancerous stage and extends throughout the full thickness of the epithelium with marked changes in the cellular characteristics, including the loss of polarity, abnormal shape, nuclear abnormalities with increased nucleus-to-cytoplasm ratios, and branched and crowded crypts of the tissues. High-grade dysplasia is associated with the highest risk of progression to EAC, greater than 5% annually, while nondysplastic BE (NDBE) is associated with relatively low annual rates of progression (0.12%–0.4%) [21–23]. Studies have provided widely varying annual rates of neoplastic progression in patients with LGD, making it difficult to estimate true annual risk. One meta-analysis of 24 studies found the annual incidence rates of progression to EAC to be 0.54% (95% CI, 0.32–0.76) and HGD/EAC to be 1.73% (95% CI, 0.99–2.47) in patients with BE-LGD [24]. BE can be further defined as short-segment or long-segment if the metaplasia involves < 3 cm or ≥ 3 cm of the esophageal mucosa, respectively [25]. The length of the segment involved is an additional predictor of neoplastic progression in BE, with one study finding the odds ratio (OR) of neoplastic progression to be 1.21 for every 1 cm of BE segment (95% CI, 1.08–1.37; *P* = 0.001) [26]. The chronic insult of the stratified squamous epithelium in the distal esophagus and EGJ induce genetic and epigenetic changes in the epithelial cell lineage. The accumulation of genetic mutations and epigenetic alterations drives the metaplasia-dysplasia-carcinoma sequence. In this process, single nucleotide variations in several genes occur with a complex and abnormal genetic architecture of the epithelium [27–33]. The architecture of the mucosal layer in BE resembles that of the small intestine with significantly increased density of goblet-like or columnar-shaped mucus secreting goblet cells. The abnormal epithelial cells in the distal esophagus and EGJ express a variety of mucin (MUC) genes, including MUC1, MUC2, MUC3, and MUC5. Additionally, the cells contain a group of secreted peptides—Trefoil Factor Family (TFF) members—including TFF1 (pS2), TFF2 (Spasmolytic polypeptide), and TFF3 (Intestinal Trefoil Factor). These peptides are primarily found in mucous epithelia in the gastrointestinal tract, protect epithelial integrity, and regulate cell migration, proliferation, and apoptosis during the repair of damaged epithelial cells. The TFFs were initially thought to be tumor suppressors, as they can play a significant role in tumor progression and metastasis. In esophageal adenocarcinoma, elevated TFF levels serve as potential biomarkers in the progression of BE to carcinoma. However, due to their involvement in other cancers, TFFs are moderately selective and specific as biomarkers in EAC [31, 34–37].

**Figure 1:**
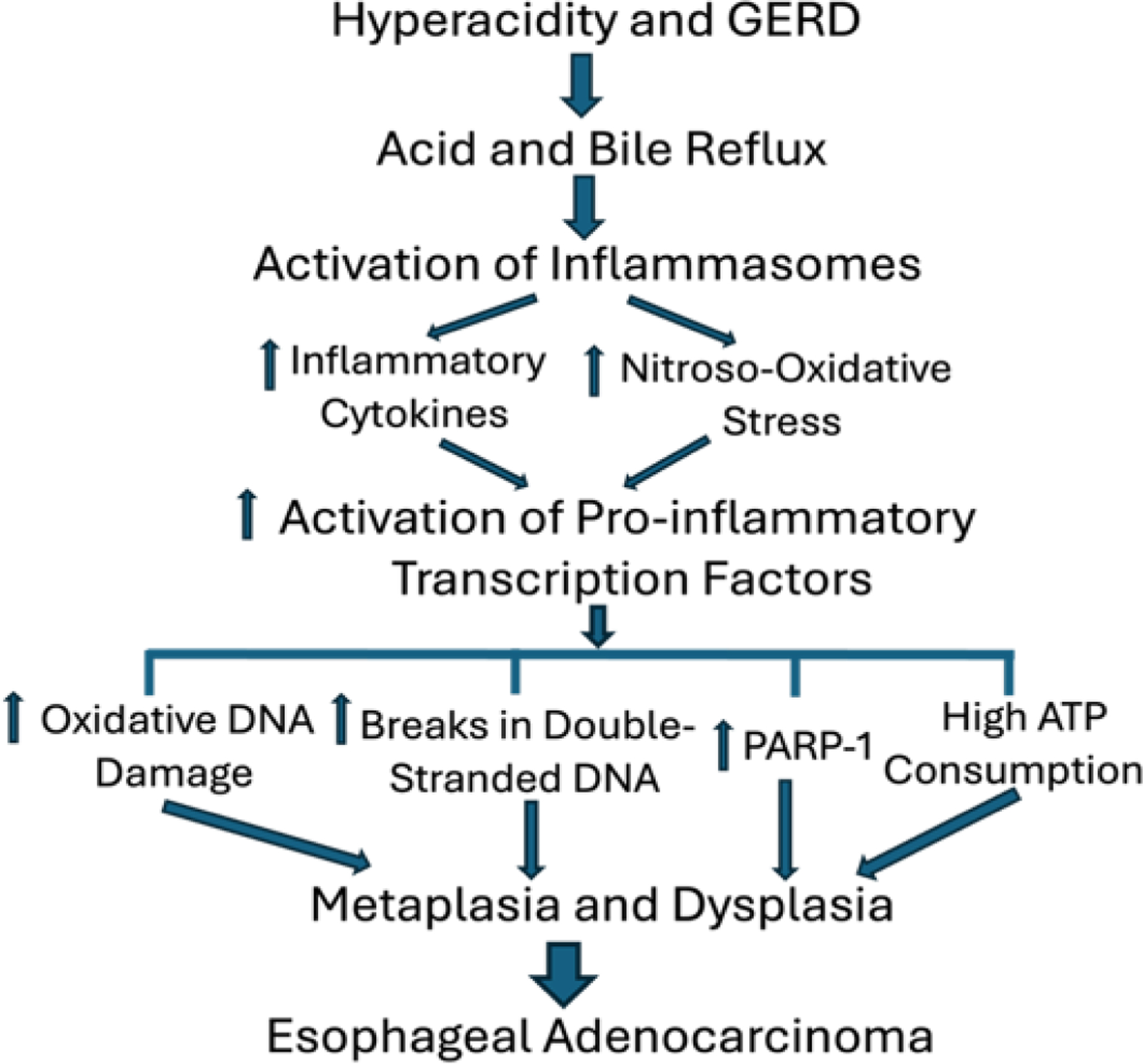
Schematic diagram showing the initial insults and chronic acid and bile reflux in the lower esophagus and at the esophagogastric junction that lead to the activation of inflammasomes and downstream cellular and molecular events in the pathogenesis of metaplasia and dysplasia leading to esophageal adenocarcinoma. GERD, gastro-esophageal reflux disease; PARP-1, poly [ADP-ribose] polymerase 1.

Two major theories, Transdifferentiation and Transcommitment, have been put forward in the acid and bile reflux-induced metaplastic changes [31,35]. In the transdifferentiation theory, the epithelial cell injury due to chronic reflux of acid and bile activate pro-inflammatory events within the esophageal cells, activating inflammasomes to produce inflammatory cytokines, activation of nitroso-oxidative stress releasing reactive oxygen species and reactive nitrogen species, followed by the activation of pro-inflammatory transcription factors. These intracellular chain of events results in increased oxidative DNA damage, double strand DNA breaks, upregulation of poly(ADP-ribose) polymerase (PARP)-1, and high consumption of ATP, leading to metaplasia and dysplasia in the distal esophagus and at the gastro-esophageal junction (Figure 1). In this process, several molecules are overexpressed, including SMAD (suppressors of mothers against decapentaplegic) and CDX2 (caudal type homeobox 2, a homeobox transcription factor protein) that work together to regulate intestinal development and differentiation, and others. In the transcommitment theory, there could be proteolytic cleavages by ADAM (a disintegrin and metalloproteinase) and g-secretase enzymes, releasing Notch intracellular domain to translocate to the nucleus and binding with a repressor complex, converting it into an activator complex, and thus regulating the transcription of target genes involved in the reprogramming of pluripotent stem cells in the submucosal esophageal glands and differentiation into columnar epithelium. Alternatively, or in addition, the residual embryonic stem cells might undergo re-programming in the esophagus [31,34,36,37].

Overall, the neoplastic progression of the BE mucosa is a complex transformation leading to polygenic multifactorial adenocarcinoma involving micro-variations in the genetic code of many genes. The molecular changes due to genetic and epigenetic changes promote uncontrolled cell growth, resistance to apoptosis, and genomic instability, progressing to the development of adenocarcinoma. Many genes are involved in cellular and molecular pathways in the underlying pathogenesis of EAC. Briefly, the *FOXF1* gene has been linked to BE and promotes a columnar phenotype; specific genetic variants of *FOXP1* are associated with increased risk for EAC; *MGST1* gene variants increase risk for BE and EAC. The *MGST1* gene encodes microsomal glutathione S-transferase 1 and protects epithelial cell membrane from oxidative stress. Single nucleotide polymorphisms in this gene decreases its anti-oxidative effects. Mutation in TP53 tumor suppressor gene occurs early and is critical in identifying the boundaries between the nondysplastic and dysplastic BE and EAC [35–37]. Gradual accumulation of mutation and alterations in *SMAD4* gene has also been reported in the progression of dysplasia to esophageal adenocarcinoma [38]. Rapid amplification of oncogenes may also occur due to genomic catastrophe involving chromothripsis (chromosomal shattering and reassembly) or breakage-fusion-bridge cycles. Indeed, genomic doubling followed by catastrophic chromosomal instability has been reported in esophageal adenocarcinoma with *TP53* mutations [39]. The *CDKN2A* gene *(p16INK4a),* which codes for the p16 cell cycle regulator, is frequently inactivated via mutation, deletion, or epigenetic silencing in BE and EAC. The inactivation of *CDKN2A* gene results in uncontrolled cellular proliferation and tumor progression. In the later stages of dysplasia and esophageal adenocarcinoma, significant amplification of oncogenes, including *ERBB2* (HER2) results in the overexpression of *HER2*. Hypermethylation of *ESR1 (Estrogen Receptor 1)* is observed at a high frequency in inflammatory reflux esophagitis and in all subsequent stages of progression [40]. *Secreted Frizzled-Related Protein family genes, SFRP1* and *SFRP5*, are frequently hypermethylated in BE and EAC [41]. The silencing of *ID4 (Inhibitor of Differentiation 4)* via methylation is another mechanism implicated in the progression of BE to EAC [42]. *TIMP3 (Tissue Inhibitor of Metalloproteinase 3)* has been found to be associated with the onset of BE metaplasia and continues to be hypermethylated through progression to EAC [43,44]. *TFPI2 (Tissue Factor Pathway Inhibitor 2)* gene is significantly hypermethylated in patients with BE compared to those with reflux [45]. Other genes include *MYUO18B, SEMA5A, CDH1 (E-cadherin), CTNNB1, APC (Adenomatous Polyposis Coli), GPX3,* and *ARID1A* [31,35,46–53].

Epigenetic changes are heritable modifications affecting gene expression without alteration in DNA sequence. Epigenetic factors primarily relate to DNA methylation, histone modification, and non-coding mRNAs. These factors are activated and serve as super-enhancers in the initiation of low-grade to high-grade dysplasia and progression to EAC. Hypermethylation of DNA results in the downregulation of oncosuppressor genes resulting in uncontrolled cellular proliferation and progression to EAC [28–35]. Histone modification includes acetylation, methylation, and ubiquitination. Aberrant histone modifications involve the histone modifying enzymes and tri-methylation of lysine 27 on histone H3 protein (H3K27me3). This leads to the formation of heterochromatic regions that downregulate nearby tumor suppressor genes, resulting in the pathogenesis of BE and progression to EAC. Histone acetylation promotes gene transcription by relaxing chromatin structure, and deacetylation compacts it resulting in gene silencing. Histone methylation can activate or repress gene transcription. Targeting lysine and arginine residues on histones in the process of ubiquitination, regulates chromatin and genome stability. Although the promoter hypermethylation is the most common underlying mechanism, the inactivation of regulatory genes could also be due to somatic mutations and loss of heterozygosity [28,29,31,35,54].

Epigenetic alterations have been reported in several genes in the precancerous lesions in the distal part of the esophagus and EGJ. This include hypermethylation of *CDKN2A* (cyclin dependent kinase inhibitor 2A) in the early phase of BE, resulting in the inactivation of *CDKN2A* activity, yielding it unable to stabilize tumor suppressor protein p53 or control cell cycle progression in the G1 phase [55]. Many additional hypermethylated genes have been reported in case of BE and EAC [31,35,56–61]. These include *MGMT (O-6-methylguanine-DNA methyltransferase* involved in DNA repair), *CDH1* and *APC* (involved in cell adhesion), *DAPK1* (involved in apoptosis), and many others including *RUNX3, TFPI, VIM, CCNA1, TWIST1, ZNF354, ZNF569, CTNND2, CCL20, p16, HPPI, NELLI, TACI, SST, ALAP12, CDH13, SLC22A18, PIGR, GIA12,* and *RIN2* [31,35,62–65]. Widespread hypomethylation in the early stage of BE metaplasia results in the activation of oncogenes and increased genomic instability [62–65].

Altered microRNA (non-coding RNAs) expression profiles have been observed in the epigenetic regulation of the underlying pathogenesis of BE and EAC [66–68]. The reduced expression of miR-192 and miR-203 has been linked to greater progression of BE to EAC [56,58,61,69,70]. However, increased expression of miR-194 and miR-215 in BE and downregulation of miR-215 in EAC have been implicated in the metaplasia and neoplastic progression to EAC and could be used as biomarkers [56,61,69]. Decreased expression of miR-205 in BE and EAC could be related to disordered of epithelial-mesenchymal transition [35,56,58,61,66,69,70].

### 2.3. Screening for Barrett’s Esophagus

The American Gastroenterological Association (AGA) and American College of Gastroenterology (ACG) recommend endoscopic screening for BE in patients with multiple risk factors, including male sex, age > 50 years, history of chronic GERD, obesity, tobacco smoking, and family history of BE or EAC in a first-degree relative [71,72].

The gold standard to screen for Barrett’s esophagus is esophagogastroduodenoscopy (EGD) using high-definition white light endoscopy (HD-WLE) and virtual chromoendoscopy (VC) along with diagnostic tissue biopsy according to the Seattle biopsy protocol (4-quadrant biopsies every 1–2 cm and target biopsies from any visible lesion) [71–74]. HD-WLE provides better characterization of early mucosal changes, and the addition of chromendoscopy has been shown to increase the detection rate of HGD/EAC versus HD-WLE alone (14.7% vs 10.1%; relative risk [RR] = 1.44) [75]. Unsedated transnasal endoscopy (uTNE) is an alternative endoscopic procedure for the screening and surveillance of BE, which can be performed in an ambulatory setting during a clinic visit. Studies have provided evidence that uTNE is safe, accurate, and well-tolerated; however, its implementation into regular use in the United States has been slow, possibly due to lower insurance reimbursement or the need for additional physician training [76]. Nonendoscopic, swallowable cell-collection devices such as the EsoCheck, EsophaCap, and Cytosponge are generally accepted as alternative screening methods [71,72]. These devices are of particular interest given their minimally invasive administration, which does not require sedation and can be done in an ambulatory setting.

### 2.4. Diagnosing Barrett’s Esophagus

The diagnosis of BE is made via endoscopic identification of salmon-colored columnar metaplasia ≥1 cm in length proximal to the esophagogastric junction based on the Prague criteria, as well as the presence of intestinal metaplasia characterized by intestinal-type goblet cells on histological examination [72,77]. Dysplasia is also assessed and further characterized as negative, low-grade, or high-grade based on the degree of nuclear enlargement, elongation, hyperchromasia, pleiomorphism, and stratification, the nucleus-cytoplasm ratio, the presence of increased/abnormal mitoses, and the loss of nuclear polarity on histologic examination [78]. Compared to low-grade dysplasia, tissues with high-grade dysplasia have a greater degree of cytologic atypia with more severe cellular, nuclear, and architectural abnormalities.

### 2.5. Treatment and Surveillance of Barrett’s Esophagus

All patients diagnosed with BE—symptomatic and asymptomatic—should be treated with long-term proton pump inhibitor (PPI) therapy such as omeprazole, esomeprazole, pantoprazole, or lansoprazole [79]. PPIs irreversibly inhibit the H+/K+ ATPase pump in gastric parietal cells, thereby reducing gastric acid secretions [80]. The ACG and AGA recommend at least once daily PPI therapy, with consideration for twice-daily dosing where clinically appropriate [71,72]. While PPIs can greatly reduce reflux-related symptoms, their chemopreventive role has long been debated. The Aspirin and Esomeprazole Chemoprevention in Barrett’s metaplasia Trial (AspECT), a large-scale randomized controlled trial, evaluated outcomes and progression between patients treated with low-dose (20 mg twice daily) and high-dose (40 mg twice daily) PPI therapy, both with and without the addition of aspirin, for over 20,000 patient years [81]. The study found that high-dose PPIs significantly increased the length of time to outcome (all-cause mortality, HGD, EAC) versus low-dose PPI (time ratio [TR] = 1.27; 95% CI, 1.01–1.58; *P* = 0.038). The effects of aspirin appeared to be additive to the effects of PPIs; however, no significant difference was found in the primary analysis between aspirin versus no aspirin (TR = 1.24; 95% CI, 0.98–1.57; *P* = 0.068). Despite the proposed additive benefits of aspirin hypothesized in this study, the AGA and ACG do not recommend routine use of aspirin for chemoprevention in patients with BE [71,72]. A multicenter prospective cohort study of 540 patients with BE found no significant effect of histamine-2 receptor antagonists on neoplastic progression, but noted significant reduction in neoplastic progression in patients using PPIs at inclusion of the study (hazard ratio [HR] = 0.41; 95% CI, 0.18–0.93) and at follow up (HR = 0.21; 95% CI, 0.07–0.66) [82]. Additionally, a meta-analysis of 7 observational studies found that PPI use was associated with a 71% reduction in risk of EAC or HGD in patients diagnosed with BE (adjusted OR = 0.29; 95% CI, 0.12–0.79) [83]. Other studies have refuted the chemopreventive effects of PPI and EAC. One such meta-analysis, comprising 9 observational studies, found no association between PPI use and risk of EAC or HGD in patients with BE (unadjusted OR = 0.43, 95% CI, 0.17–1.08) [84].

Patients diagnosed with NDBE on endoscopy are recommended to undergo repeat surveillance endoscopy in 3 to 5 years [71,72]. The ACG supports consideration of segment length when determining an appropriate surveillance protocol, such that segments of NDBE <3 cm are assigned 5-year surveillance intervals while segments of NDBE ≥3 cm are assigned 3-year intervals [72]. Biopsy protocols for surveillance endoscopy are like those utilized during screening, most commonly using the Seattle protocol [74].

The management of patients diagnosed with LGD continues to be debated. The ACG recommends endoscopic eradication therapy with radiofrequency ablation (RFA) to reduce the risk of progression to HGD but notes that endoscopic surveillance is an acceptable alternative [72]. When the latter route is taken, the ACG recommends surveillance endoscopies every 6 months for 1 year, then annually thereafter. If no dysplasia is seen during endoscopy, intervals may be increased to every 3 years.

Severe erosive esophagitis (EE) of Los Angeles Grade B or worse can mask dysplasia on endoscopic examination. One prospective study of 172 patients diagnosed with EE found that 12% of patients were diagnosed with BE on repeat examination after undergoing standard acid-suppression therapy with proton-pump inhibitors (PPIs) [85]. Thus, patients diagnosed with erosive esophagitis on an initial screening endoscopy are advised to repeat endoscopic examination after completing an 8-week course of PPI treatment [72].

### 2.6. Diagnosing Esophageal Adenocarcinoma

The vast majority of EAC diagnoses are made outside of structured surveillance programs, with >75% of new cases diagnosed in advanced stages [86]. In fact, nearly 40% of patients are categorized as stage IV upon initial EAC diagnosis [2]. Most patients present with rapidly progressive dysphagia for both solids and liquids. They may also present with unplanned weight loss, fatigue, and rarely iron-deficiency anemia. Diagnosis is made via EGD with HD-WLE, VC, and tissue biopsy utilizing the Seattle protocol. EAC may be endoscopically identified based on nonspecific mucosal changes, in addition to strictures, ulcers, nodules, or masses. Superficial, early neoplasms are more difficult to visualize, with approximately 40% of early cancers diagnosed via the 4-quadrant biopsy protocol [87]. Histopathologic assessment of the endoscopic biopsies are performed and reported based on the World Health Organization (WHO) criteria [88].

### 2.7. Cancer Staging

After an established diagnosis of EAC is made, patients undergo pretreatment clinical staging to evaluate the extent of disease and to rule out distant metastases. Staging is based on the American Joint Committee on Cancer (AJCC) TNM system, with T denoting the size of the tumor, N denoting the spread to nearby lymph nodes, and M denoting the presence or number of distant metastases (Table 1) [89]. Pretreatment tumors are given a clinical stage (cTNM) based on the results of various imaging studies and diagnostic tests, while a pathological stage (pTNM) is typically assigned postoperatively based on direct sampling of the surgical tissue. A tumor grade is also provided, ranging from G1-G3 based on the level of cellular differentiation when viewed microscopically.

Adenocarcinoma of the EGJ will also be assigned a Siewert classification (Table 2) [90]. The AJCC 8th edition classifies Siewert Type I and II as esophageal cancer, while Siewert Type III was classified as gastric cancer [89].

Endoscopic ultrasound (EUS), contrast-enhanced computed tomography (CT), and whole-body integrated fluorodeoxyglucose (FDG) positron emission tomography (PET)/CT are key components of initial clinical pretreatment staging [91]. Various studies have demonstrated the clinical utility of each exam individually [92–96]. EUS is more clinically useful for staging locoregional disease and provides superior T staging versus CT or FDG-PET [92]. FDG-PET/CT is the most sensitive imaging modality for detecting distant metastatic disease (M staging), and its superiority over CT in detecting occult lesions may prevent unnecessary exploratory surgery [93]. In one prospective multi-center trial, the addition of FDG-PET to clinical staging resulted in significant impacts on treatment decisions in 38% of patients, detecting additional lesions or sites of disease in 41% of patients [94]. However, FDG-PET/CT is ineffective and possibly detrimental when utilized in early-stage disease, often leading to additional, unnecessary biopsies [97]. The addition of IV contrast for CT imaging is necessary for diagnosing some small metastases—a protocol uncommonly used in standard FDG-PET/CT imaging [98]. Thus, IV contrast CT is an important, complementary diagnostic test in the pretreatment staging process. Bronchoscopy is recommended for tumors located at or above the carina (tracheal bifurcation) to assess invasion into the tracheobronchial tree [91,99]. Staging laparoscopy is warranted in some cases to assess peritoneal involvement, especially for advanced or node-positive tumors with concern for M1 disease and for tumors located at the EGJ (Siewert type II and III) [91,100].

Routine labs, such as a complete blood count (CBC) and comprehensive metabolic profile (CMP), are ordered to rule out metabolic or hematologic disorders and to establish a baseline by which to compare throughout treatment. Additional screening tests of biological specimens may be performed to guide treatment decisions. The National Comprehensive Cancer Network (NCCN) recommends universal testing for microsatellite instability (MSI) by polymerase chain reaction (PCR)/next generation sequencing (NGS) or mismatch repair deficiency (dMMR) by immunohistochemistry (IHC) on formalin-fixed paraffin-embedded (FFPE) tissue in all patients with newly diagnosed esophageal or EGJ adenocarcinoma [91]. Biomarkers including Claudin 18 isoform 2 (CLDN18.2), tumor human epidermal growth factor receptor 2 (HER2) amplification, programmed death-ligand 1 (PD-L1) protein expression, tumor mutational burden (TMB), neurotrophic-tropomyosin receptor kinase (NTRK) gene fusion, and rearranged during transfection (RET) gene fusion may also be screened for to guide treatment decisions, most commonly in the setting of inoperable locally advanced, metastatic, or recurrent cancers.

The Subjective Global Assessment (SGA) is a commonly used screening tool for determining nutritional status [101]. Patients are given a grade of A (well-nourished), B (moderately/suspected of being malnourished), or C (severely malnourished). Evaluating a patient’s nutritional status is an essential aspect of the pretreatment assessment, as malnutrition is strongly correlated with poorer surgical outcomes, post-treatment decline, and a lower quality of life [102,103]. A multidisciplinary treatment approach—one which includes dietitian involvement early in the pretreatment staging—is fundamental for improving long-term outcomes [104]. Additional lifestyle modifications such as smoking cessation is strongly advised to all newly diagnosed patients.

## 3. Treatment of Esophageal Adenocarcinoma

### 3.1. Overview

The selected treatment for EAC depends upon clinical staging, but may vary based on a patient’s age, their suitability for surgery, tumor markers, medical contraindications, and patient preference. Endoscopic eradication treatments, such as endoscopic mucosal resection (EMR), radiofrequency ablation (RFA), and photodynamic therapy (PDT), are typically reserved for superficial cancers (Tis, T1a) in individuals with low risk of lymph node metastasis [91,105,106]. This approach normally requires frequent, lifelong post-operative surveillance for recurrence.

**Figure 2:**
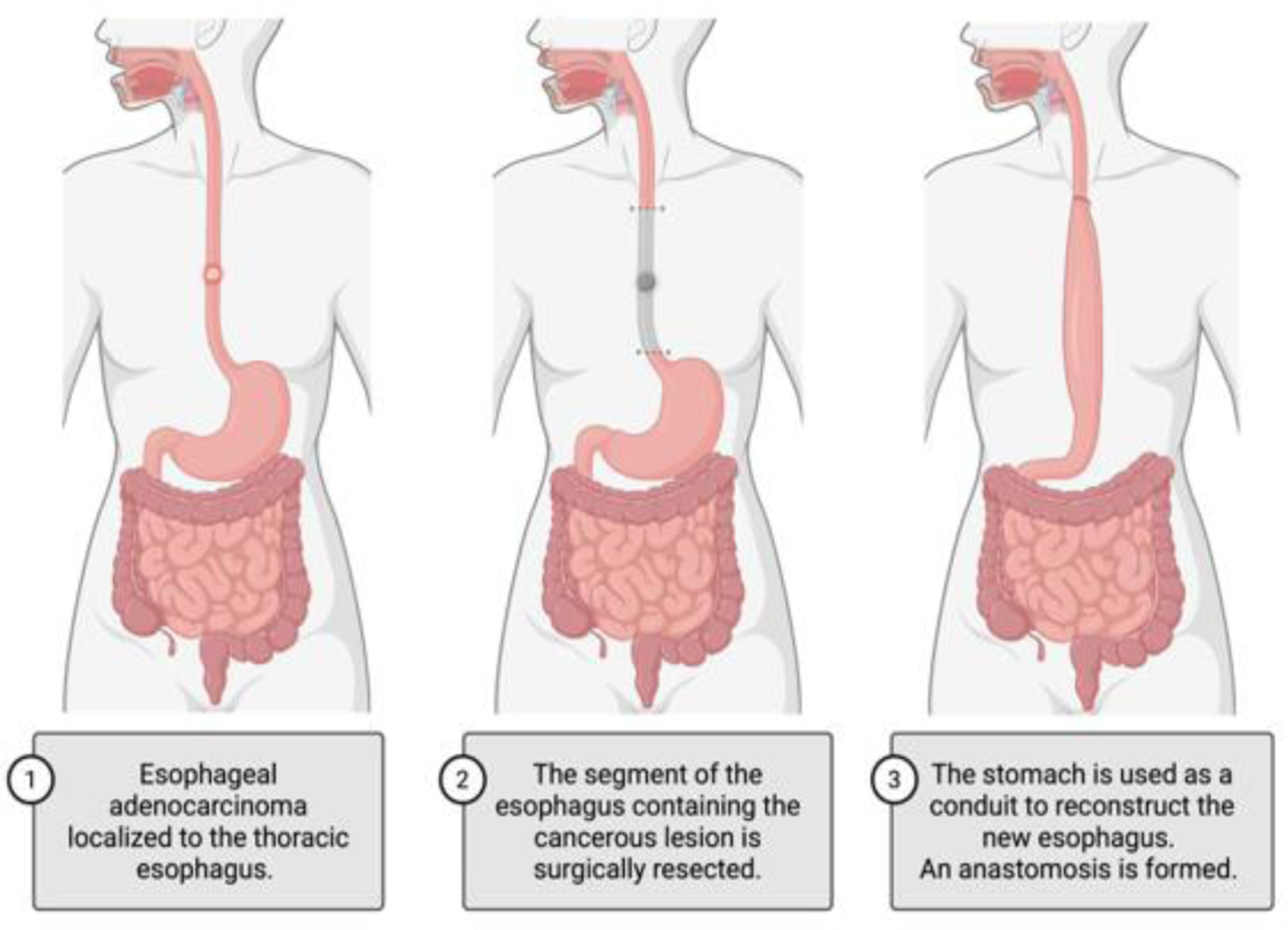
Esophagectomy to surgically remove the cancerous lesion followed by reconstruction of new esophagus.

Alternatively, esophagectomy, a surgical procedure removing all or part of the patient’s esophagus, is a definitive treatment option that can be utilized independently or as a part of a multi-modal approach (Figure 2). There are varying approaches to esophagectomy depending on the tumor location and size, and any history of radiation or surgical intervention. Approaches include trans hiatal esophagectomy (THE) and transthoracic esophagectomy (TTE) [107,108]. THE involves midline laparotomy and left cervical incision with cervical anastomosis. TTE most commonly involves laparotomy with right thoracotomy followed by intrathoracic anastomosis (Ivor Lewis) but can also be performed via a three-incision approach (thoracotomy, laparotomy, cervical incision) with cervical anastomosis (McKeown) [108]. NCCN recommendations for patients undergoing esophagectomy include the removal of at least 15 regional lymph nodes for testing, the use of a gastric conduit for reconstruction of the esophagus, and surgical execution within a high-volume center, where at least 15 to 20 esophageal procedures are performed annually [91].

Based on the most recent data from the Esophagectomy Complications Consensus Group (ECCG), the incidence of complications with esophagectomy is 59%, with the most common being pneumonia (14.6%), atrial dysrhythmia (14.5%), infection (14.2%), and anastomotic leak (11.4%) [109]. Advancements in minimally invasive surgery (MIS) over the last decade have drastically changed the field of surgical oncology, as open surgery is associated with significant morbidity and mortality [110]. Despite this, there is no apparent consensus for utilizing minimally invasive esophagectomy (MIE) versus traditional open esophagectomy (OE). A retrospective review queried from The Society of Thoracic Surgeons (STS) National Database (v2.081) compared surgical outcomes of MIE vs OE using 3,780 esophageal resections [111]. They found equivalent rates of morbidity and all-cause mortality between MIE and OE cases. MIE was found to have longer median procedure times (443 vs 312 minutes; *P* <0.001), shorter median length of hospital stay (9 vs 10 days; *P* < 0.001), and higher rates of reoperation (9.9% vs 4.4%; *P* < 0.001). Open resection was associated with higher rates of wound infection (6.3% vs 2.3%; P < 0.001), postoperative transfusion (18.7% vs 14.1%; *P* = 0.002), and ileus (4.5% vs 2.2%; *P* = 0.002). A later meta-analysis of 14,311 cases of resectable esophageal cancer across 48 studies also compared outcomes between MIE and OE [112]. These researchers found that MIE was associated with a significant reduction of in-hospital mortality compared to open esophagectomy (OR = 0.69; 95% CI, 0.55–0.86), significantly reduced incidence of pulmonary complications (RR = 0.73; 95% CI, 0.63–0.86), pulmonary embolism (OR = 0.71; 95% CI, 0.51–0.99) and arrhythmia (OR = 0.79; 95% CI, 0.68–0.92). No significant difference in the occurrence of anastomotic leak (OR = 0.93; 95% CI, 0.78–1.11) or gastric tip necrosis (OR = 0.89; 95% CI, 0.54–1.49) was seen between groups.

An additional retrospective analysis utilizing 14,880 patients with 4,572 propensity-matched pairs from 2014–2017 found that MIE was associated with lower incidences of in-hospital mortality (1.2% vs 1.7%; *P* = 0.048), surgical site infection (1.9% vs 2.6%; *P* = 0.04), anastomotic leakage (12.8% vs 16.8%; *P* < 0.001), blood transfusion (21.9% vs 33.8%; *P* < 0.001), reoperation (8.6% vs 9.9%; *P* = 0.03), tracheotomy (4.8% vs 6.3%; *P* = 0.002), and unplanned intubation (6.3% vs 8.4%; *P* < 0.001) [113]. Compared to OE, patients who underwent MIE had shorter post-operative hospitalization (23 vs 26 days; *P* < 0.001), but a longer duration of anesthesia (408 vs 363 minutes; *P* <0.001), prolonged post-operative intubation (23.2% vs 19.3%; *P* <0.001), and higher incidences of vocal cord dysfunction (9.2% vs 7.5%; *P* <0.001).

## 4. Treatment Based on Clinical Stage (cTNM)

### 4.1. cTis

The earliest clinical stage of EAC, also referred to as high-grade dysplasia, can be treated with endoscopic eradication with lifelong surveillance or definitive esophagectomy [91,114].

### 4.2. cT1N0

Most T1 tumors are initially treated with esophagectomy [114]. In cases of positive surgical margins, this can be followed by chemoradiotherapy (CRT). T1a tumors may be treated with EMR followed by RFA. Studies have highlighted similar efficacy between surgical resection versus endoscopic treatments, with comparable rates of overall survival (OS) and mortality [115]. In a more recent propensity matched study using 735 patients with T1a EAC from the National Cancer Database, endoscopic resection was associated with shorter hospitalization, fewer readmissions, and lower 90-day mortality (HR = 0.15; *P* = 0.003). However, surgical resection proved superior to endoscopic resection in mortality rate in patients surviving greater than 90 days (HR = 1.34; *P* = 0.02) [116].

### 4.3. cT2N0

The best treatment approach for cT2 tumors in the absence of positive lymph nodes is under debate. Some advocate for the same treatment protocols as more advanced tumors (cT3–4 or N+), while others endorse initial surgical resection [114,117,118].

### 4.4. cT3-T4a or N+

A multimodal approach is recommended for locally advanced, surgically resectable esophageal and EGJ adenocarcinoma [119]. Even when negative surgical margins (R0) are achieved, surgical resection alone cannot typically address the highly aggressive nature of more advanced tumors [120]. Perioperative chemotherapy (periCTX) with fluorouracil (5-FU), leucovorin, oxaliplatin, and docetaxel (FLOT) plus surgical resection is generally recommended as the first line of treatment [121]. For individuals with contraindications to chemotherapy (CTX) (i.e., comorbidities, older age, intolerance, etc.), neoadjuvant chemoradiation (neoCRT) with carboplatin, paclitaxel, and concurrent radiotherapy (CROSS) followed by surgery is an appropriate alternative. NeoCRT may be followed by adjuvant immunotherapy for residual disease after surgical resection. The ESOPEC trial (randomized, multicenter, phase 3 trial) compared treatment outcomes of periCTX with FLOT plus surgery versus neoCRT (CROSS protocol) plus surgery for locally advanced esophageal and EGJ adenocarcinoma (primary tumor cTNM of cT1 cN+, cT2–4a cN+, or cT2–4a cN0) [122]. The study found that periCTX with FLOT resulted in greater progression-free survival (PFS) at three years (51.6%) compared to the neoCRT group (35.0%) and greater OS (57.4% vs 50.7%) at three years (HR = 0.70; 95% CI, 0.53–0.92; *P* = 0.01). Definitive CRT (dCRT) is an alternative for those not suitable for surgery [123]. All patients with advanced disease are advised to enroll in clinical trials when available. Details regarding the various clinical trials for the treatment of locally advanced, surgically resectable tumors are summarized below.

### 4.5. Perioperative Chemotherapy

The FLOT-4 AIO trial, compared OS between adjuvant and neoadjuvant docetaxel-based chemotherapy (FLOT) plus surgery versus epirubicin, cisplatin, and 5-FU (ECF/ECX; control group) plus surgery in patients with clinical stage cT2 or higher and/or node-positive (cN+) gastric or EGJ adenocarcinoma [124]. They found that FLOT was associated with a higher proportion of pathological complete response (pCR) compared to ECF/ECX (16% vs 16%, *P* = 0.02). Median OS was increased in the FLOT group (50 months) compared with the ECF/ECX group (35 months) (HR = 0.77; 95% CI, 0.63–0.94). The 3-year OS rate was 48% with ECF/ECX and 57% with FLOT. The most common non-surgical adverse effects amongst the FLOT group were neutropenia (52%), leukopenia (28%), nausea (9%), infection (12%), fatigue (9%), and vomiting (3%).

For patients who are unlikely to tolerate the FLOT regimen, oxaliplatin, 5-FU, and leucovorin (FOLFOX) is an acceptable alternative [125]. The major differences between FLOT and FOLFOX lie in the administration of the 5-FU and the addition of docetaxel in FLOT. In the FLOT protocol, 5-FU is administered via 24-hour infusion. For the FOLFOX protocol, 5-FU is administered via IV bolus followed by up to 46–48 hours of infusion. The FOLFOX regimen requires patients to carry a continuous, portable infusional pump, which may be a deterrent for some patients. The CALGB 80803 (Alliance) Trial represented the efficacy of FOLFOX for periCTX, comparing treatment responses via PET scan against paclitaxel plus carboplatin [126]. Among the FOLFOX PET responders, 40.3% achieved pCR (95% CI, 28.9–52.5) versus 14% carboplatin/paclitaxel PET responders (95% CI, 6.6–25.0), and a 5-year survival of 53 months (95% CI, 42.5–66.1) versus 43.9 months (95% CI, 33.1–58.2), respectively.

### 4.6. Neoadjuvant Chemoradiotherapy

The CROSS trial compared neoCRT plus surgery versus surgery alone for esophageal or esophagogastric cancers [127,128]. Patients with squamous-cell carcinoma (23%), adenocarcinoma (75%), large-cell undifferentiated carcinoma (2%), as well as cancer of the gastric cardia with clinical stage cT1N1 or cT2–3N0–1 were included in the study. The neoCRT group was treated with weekly carboplatin and paclitaxel for 5 weeks and concurrent RT (41.4 Gy in 23 fractions, 5 days per week) followed by surgical resection. OS was greater in the neoCRT group vs surgery alone (HR = 0.66; 95% CI, 0.50– 0.87; *P* = 0.003). Negative surgical margins (R0) were achieved in 92% of the neoCRT cohort versus 69% in the surgery alone (*P* <0.001). Postoperative complications were similar between the two cohorts. The most common hematologic and non-hematologic adverse effects associated with CRT were leukopenia (6%), neutropenia (2%), anorexia (5%), and fatigue (3%).

FOLFOX may also be used as an alternative radiosensitizing regimen for neoCRT and dCRT, with various studies highlighting its potential comparability to carboplatin/paclitaxel [129].

### 4.7. Adjuvant Therapy

The best choice for second-line systemic therapy for local recurrence or residual disease following surgical resection is highly dependent on initial treatment. The development of molecular-targeted agents has greatly altered standard of care for residual and recurrent disease. Nivolumab, a programmed death receptor-1(PD-1)-blocking antibody, is often considered in patients with residual disease after neoCRT and surgical resection [91,130]. Ramucirumab, a human vascular endothelial growth factor receptor 2 (VEGFR2) antagonist, is also utilized as a second line therapy for patients with disease progression on or after prior fluoropyrimidine- or platinum-containing chemotherapy [91,131,132]. Their respective clinical trials and indications will be further discussed under the subheading “Molecular-Targeted Treatments.”

Adjuvant chemotherapy may also be utilized for patients not achieving pCR following neoadjuvant CRT and surgical resection. While multiple options exist, most experts recommend choosing different agents for adjuvant therapy than those utilized during neoadjuvant therapy [91]. Monotherapy with docetaxel, paclitaxel, or irinotecan are NCCN preferred options for second-line or subsequent therapy in the setting of residual disease or recurrence [91]. The COUGAR-02 phase III trial demonstrated the OS benefit of utilizing single-agent docetaxel for recurrent disease versus symptom management alone (5.2 vs 3.6 months; HR = 0.67; *P* = 0.01) [133]. The WJOG 4007 phase III trial compared utilization of single agent paclitaxel vs irinotecan for second-line therapy, finding similar OS between the two groups (9.5 vs 8.4; HR = 1.13; *P* = 0.38) [134]. FOLFIRI (leucovorin, fluorouracil, and irinotecan) was shown to be an efficacious second-line option in a cohort study of patients with gastric of EGJ adenocarcinoma refractory to docetaxel (OS = 6.2 months; PFS = 3.8 months) [135]. Other options for second-line therapy include combination therapy with irinotecan and cisplatin, ramucirumab with irinotecan and with or without 5-FU, and docetaxel with irinotecan [136–138]. The TAGS phase III trial demonstrated improved median OS for patients refractory to at least 2 prior chemotherapy regimens when treated with trifluridine/tipiracil and best supportive care compared to placebo and best supportive care (5.7 vs 3.6 months; HR = 0.69; 95% CI, 0·56–0·85; *P* = 0.00029) [139]. Grade 3 or worse adverse events occurred in 80% of the trifluridine/tipiracil group compared to 58% in the placebo group.

## 5. Treatment for Unresectable Tumors

Esophageal tumors with clinical stage cT4b invade unresectable adjacent structures (i.e., aorta, vertebral bodies, trachea) and thus are very rarely treated surgically [140,141]. Esophageal tumors of the cervical region are rarely surgically resected, given their close approximation to major organs (larynx, pharynx, thyroid) and risk for functional impairment and poor quality of life [142,143]. Patients with distant metastatic disease are not typically candidates for surgical resection and are normally referred for palliative care. Definitive CRT is the gold standard for locally advanced, unresectable EAC [91,144]. Standard of care is paclitaxel and carboplatin with concurrent RT at a dose of 50.4 Gy delivered in 28 fractions [145]. Various trials have demonstrated that dCRT confers a greater OS compared to RT alone. The Radiation Therapy Oncology Group (RTOG) 85–01 phase III trial demonstrated the OS survival benefits of patients who received dCRT verses dRT alone (12.5 vs 8.9 months) [146]. At an extended 5-year follow up, OS was 26% in the CRT group and 0% in the RT group [147].

## 6. Molecular-Targeted Treatments

Biomarkers are not only helpful in predicting disease progression, but also in the selection of the targeted therapy based on effectiveness of the treatment. Over the last decade, ongoing research on biomarkers involved in various cancer pathways has led to the development and approval of targeted immunotherapies. While MSI and MMR screening are recommended universal tests for all patients diagnosed with esophageal or EGJ adenocarcinoma, additional biomarker testing may be recommended for patients with advanced or inoperable cancers in whom an approved treatment is available and appropriate [91].

## 7. Programmed Death Receptor-1(PD-1)-Blocking Antibodies

### 7.1. Nivolumab

Opdivo (nivolumab) is FDA approved to treat esophageal and EGJ adenocarcinoma for patients with residual pathologic disease after complete surgical resection (R0) and neoadjuvant CRT or in combination with other fluoropyrimidine- and platinum-containing chemotherapeutics for patients with advanced or metastatic esophageal or EGJ adenocarcinoma [130].

The CHECKMATE-577 trial led to the 2021 approval of Opdivo (nivolumab) for patients with residual pathologic disease after complete surgical resection (R0) and neoadjuvant CRT for esophageal or EGJ adenocarcinoma [148]. The study demonstrated a significant improvement in disease-free survival in the nivolumab group versus placebo group (HR 0.69; 95% CI: 0.56, 0.85; *P* = 0.0003), regardless of PD-L1 expression. Serious adverse events due to treatment occurred in 8% of the nivolumab group. The most common adverse effects in the nivolumab group were fatigue and diarrhea (17%).

The CHECKMATE-649 trial led to the 2021 approval of Opdivo (nivolumab), used in combination with other fluoropyrimidine- and platinum-containing chemotherapeutics, for patients with advanced or metastatic esophageal or EGJ adenocarcinoma [149]. During the study, patients were randomly assigned to nivolumab plus chemotherapy or chemotherapy alone treatment groups. PD-L1 combined positive scores (CPS) were centrally determined during the study, and the main efficacy outcomes were assessed in patients with PD-L1 CPS ≥ 5. They found a significant increase in PFS (7.7 vs 6.0 months; HR = 0.68; 95% CI, 0.58–0.79; *P* < 0.0001) as well as OS (14.4 vs 11.1 months; HR = 0.71; 95% CI, 0.61–0.83; *P* < 0.0001). The most common side effects observed (≥25%) in both groups were nausea, diarrhea, and peripheral neuropathy.

### 7.2. Tislelizumab

Tevimbra (tislelizumab-jsgr) FDA approved to treat HER2-negative, PD-L1 expressing (CPS ≥1) EGJ adenocarcinoma in combination with fluoropyrimidine- and platinum-containing chemotherapy for the first-line treatment of locally advanced unresectable or metastatic disease [150]. The RATIONALE-305 trial led to the 2025 FDA approval of Tevimbra (tislelizumab-jsgr) for gastric and EGJ adenocarcinoma [151]. During the study, patients were randomly assigned to receive tislelizumab plus chemotherapy or placebo plus chemotherapy. They found greater overall OS in the tislelizumab treatment group compared to the placebo group in patients with a PD-L1 tumor area positivity (TAP) score of ≥5% (median 15.0 vs 12.9 months; HR = 0.80; 95% CI, 0.70–0.92; *P* = 0.001). Grade 3 or worse adverse events occurred in 54% of the tislelizumab group and 50% in the placebo group.

### 7.3. Dostarlimab

Jemperli (dostarlimab-gxly) is FDA approved to treat recurrent or advanced dMMR solid tumors that have progressed on or following prior treatment and who have no satisfactory alternative treatment options [152].

### 7.4. Pembrolizumab

Keytruda (pembrolizumab) is FDA approved to treat esophageal and EGJ adenocarcinoma in patients with locally advanced unresectable or metastatic disease who meet one of the following criteria [153]:

- in combination with other fluoropyrimidine- and platinum-containing chemotherapeutics in patients not amenable to surgical resection or dCRT whose tumors express PD-L1 (CPS ≥1).
- in combination with fluoropyrimidine- and platinum-containing chemotherapy with or without the addition of trastuzumab, for the first-line treatment of HER2-positive EGJ adenocarcinoma whose tumors express PD-L1 (CPS ≥1).
- for patients with unresectable or metastatic MSI-H or dMMR solid tumors that have progressed following prior treatment and who have no satisfactory alternative treatment options.

The KEYNOTE-590 phase III clinical trial represented the clinical utility of pembrolizumab as a first line treatment in patients with advanced unresectable or metastatic esophageal or Siewert type I EGJ adenocarcinoma [154]. Patients were randomly assigned to pembrolizumab plus 5-FU and cisplatin or placebo plus 5-FU and cisplatin. OS was increased in the pembrolizumab group (12.4 months) versus placebo group (9.8 months) regardless of PD-L1 status (HR = 0.73; 95% CI, 0.62–0.86; P < 0.0001), as well as PFS (6.3 months vs 5.8 months; HR = 0.65; 95% CI, 0.55–0.76; P < 0.0001). Patients with both squamous cell carcinoma and adenocarcinoma of the esophagus were enrolled in the trial, and OS was not independently assessed for EAC. Treatment related adverse events grade 3 or higher occurred in 72% of the pembrolizumab group versus 68% in the placebo group.

The KEYNOTE-811 trial demonstrated that the addition of pembrolizumab to trastuzumab and chemotherapy resulted in greater OS (20.0 months) versus trastuzumab and chemotherapy alone (16.8 months) when used as a first line treatment for patients with unresectable or metastatic HER2-positive EGJ adenocarcinoma (HR = 0.80; 95% CI, 0.67–0.94; P = 0.004) [155]. Observed incidence of adverse effects was similar between pembrolizumab versus placebo, with the most common being diarrhea (52.5% vs 44.4%), nausea (48.8% vs 44.4%), and anemia (41.0% vs 44.0%).

## 8. Human Epidermal Growth Factor Receptor 2 (HER2)-Positive Tumors

### 8.1. Trastuzumab

Herceptin (trastuzumab), a monoclonal antibody that acts as a HER2 inhibitor, is FDA approved for the treatment of metastatic HER2 overexpressing gastric and EGJ adenocarcinoma who have not received prior treatment [156]. In the presence of locally advanced unresectable, metastatic, or recurrent HER2-positive esophageal and EGJ adenocarcinoma, the NCCN recommends fluoropyrimidine- and platinum-containing chemotherapeutics (FOLFOX or CAPOX) with the addition of trastuzumab alone or in combination with pembrolizumab if PD-L1 CPS ≥1 [91]. The ToGA phase III clinical trial compared rates of OS between patients treated with trastuzumab plus chemotherapy (capecitabine plus cisplatin or fluorouracil plus cisplatin) versus chemotherapy alone [157]. The study found a significantly greater median OS in the trastuzumab treatment group compared to the chemotherapy alone group (13.8 vs 11.1 months; HR = 0.74; 95% CI, 0.60–0.91; P = 0.0046). Rates of overall grade 3 or 4 adverse events and cardiac adverse events were similar between the two groups. The most common adverse effects in the trastuzumab group versus chemotherapy group were nausea (67% vs 63%), vomiting (50% vs 46%), and neutropenia (53% vs 57%).

For patients with recurrent HER2-positive EGJ adenocarcinoma who were initially treated with trastuzumab, Enhurtu (fam-trastuzumab deruxtecan-nxki) (a HER2-directed antibody and topoisomerase inhibitor conjugate) is a recommended second-line treatment [158]. The DESTINY-Gastric04 phase III clinical trial compared rates of OS between trastuzumab deruxtecan and ramucirumab plus paclitaxel, a common second line therapy, in patients with HER2-positive metastatic gastric or EGJ adenocarcinoma with progression following initial trastuzumab treatment [159]. The study found significantly greater median OS in the trastuzumab deruxtecan group compared to ramucirumab plus paclitaxel group (14.7 vs 11.4 months; HR = 0.70; 95% CI, 0.55–0.90; P = 0.004). The incidence of drug related adverse effects and grade 3 or higher adverse effects in the trastuzumab deruxtecan versus ramucirumab plus paclitaxel group was similar: 93% vs 91.4% and 50.0% vs 54.1%, respectively.

## 9. Claudin 18 Isoform 2 (CLDN18.2)-Directed Cytolytic Antibodies

### 9.1. Zolbetuximab

Vyloy (zolbetuximab-clzb) is FDA approved to treat CLDN18.2-positive, HER2-negative EGJ adenocarcinoma in combination with fluoropyrimidine- and platinum-containing chemotherapy for locally advanced unresectable or metastatic disease [160].

The SPOTLIGHT trial led to the 2024 FDA approval of Vyloy (zolbetuximab-clzb) [161]. Patients with CLDN18.2-positive (defined as ≥75% of tumor cells showing moderate-to-strong membranous CLDN18 staining), HER2-negative, previously untreated, locally advanced unresectable or metastatic gastric or EGJ adenocarcinoma were included in the study. Patients were randomly assigned to the zolbetuximab plus mFOLFOX6 (modified folinic acid [or levofolinate], fluorouracil, and oxaliplatin) or placebo plus mFOLFOX6 treatment groups. The study found a significant decrease in disease progression or death in the zolbetuximab treatment group compared to the placebo group (HR = 0.75; 95% CI, 0.60–0.94; P = 0.006). The most common grade 3 or worse adverse events were nausea, vomiting, and decreased appetite.

## 10. Human Vascular Endothelial Growth Factor Receptor 2 (VEGFR2) Antagonist

### 10.1. Ramucirumab

Cyramza (ramucirumab) is FDA approved to treat advanced or metastatic EGJ adenocarcinoma for patients with disease progression on or after prior fluoropyrimidine- or platinum-containing chemotherapy as a single agent or in combination with paclitaxel [131]. The RAINBOW phase III clinical trial demonstrated the clinical utility of ramucirumab as a second line chemotherapeutic for patients with metastatic gastric or EGJ adenocarcinoma with disease recurrence within 4 months of treatment with platinum- and fluoropyrimidine-based combination therapy [132]. Patients with metastatic gastric or EGJ adenocarcinoma were randomly assigned to the ramucirumab plus paclitaxel group or placebo plus paclitaxel group. Researchers found a significant increase in OS between the ramucirumab group versus placebo group, 9.6 months and 7.4 months, respectively (HR = 0.81; 95% CI, 0.68–0.96; P = 0.0169). Common (>5%) grade 3 or higher adverse events that occurred in the ramucirumab versus placebo groups included neutropenia (41% vs 19%), leucopenia (17% vs 7%), hypertension (14% vs 2%), fatigue (12% vs 5%), anemia (9% vs 10%), and abdominal pain (6% vs 3%).

## 11. Tyrosine kinase inhibitors (TKIs)

### 11.1. Repotrectinib, Larotrectinib, and Entrectinib

Augtyro (repotrectinib), Vitrakvi (larotrectinib), and Rozlytrek (entrectinib) are FDA approved to treat NTRK gene fusion-positive tumors in patients who are metastatic or where surgical resection is likely to result in severe morbidity, and have no satisfactory alternative treatments, or have progressed following treatment [162–164].

### 11.2. Selpercatinib

Retevmo (selpercatinib) is FDA approved to treat locally advanced or metastatic RET gene fusion-positive solid tumors that have progressed on or following prior systemic therapy or who have no satisfactory alternative treatment options [165].

### 11.3. Dabrafenib and Trametinib

Tafinlar (dabrafenib) and Mekinist (trametinib) are FDA approved in combination to treat unresectable or metastatic solid tumors with BRAF V600E mutation who have progressed following prior treatment and have no satisfactory alternative treatment options [166,167].

## 12. Nutritional Supplementation

Treatments to optimize nutritional status are provided perioperatively in patients with resectable disease, as well as in a palliative care setting for symptom control and improved quality of life. Nausea, dysphagia, sarcopenia, and weight loss are common in patients with EAC, often secondary to chemotherapy and/or radiation as well as cancer cachexia [168]. Esophageal cancer is associated with the greatest median weight loss before diagnosis of all cancer types, with 80% of patients affected by malnutrition [169]. Various studies have suggested that involuntary weight loss (>10%) is associated with lower 5-year survival rates, disease-specific mortality, and all-cause mortality [170,171].

For patients with esophageal cancer requiring pre-surgical nutritional supplementation, enteral nutrition is generally preferred to parenteral nutrition, as studies suggest that parenteral nutrition is associated with higher rates of infection and longer hospitalization [172,173]. While the optimal route for enteral nutrition has been long debated, the NCCN recommends preoperative placement of a jejunostomy (J) tube or esophageal dilatation for patients with severe or high risk of malnutrition [91]. Percutaneous endoscopic gastrostomy (PEG) tubes are often avoided in surgical patients to preserve the gastric conduit for reconstruction, given the risk of injury to the gastroepiploic artery during placement [91,174]. For postoperative feeding, the Enhanced Recovery After Surgery (ERAS) Society recommends early enteral feeding via J-tube, nasoduodenal (ND), or nasojejunal (NJ) tube with target nutrition rates on day 3–6 [175].

## 13. Palliative and Supportive Care

Palliative and supportive care may be provided to patients at any stage of illness and at any point of time during the delivery of care. Best supportive care is provided based on individual factors, including quality of life, symptom relief, and personal goals of care, regardless of whether cancer treatment is of curative intent. In addition to symptom control, advanced-care planning, mental health practitioners, social workers, spiritual care specialists, and care givers play an integral role in palliative care for patients with any form of cancer.

Dysphagia is one of the most common presenting symptoms in patients with esophageal cancer [176]. Palliative surgical resection may be considered in patients with severe dysphagia to improve quality of life with or without curative intent [177]. Patients with severe dysphagia who are not surgical candidates are typically treated with an esophageal stent, which has been shown to provide rapid improvement of symptoms [178]. However, modern studies have demonstrated that external-beam RT (EBRT) provides equivalent alleviation of dysphagia symptoms while providing greater pain control and lower risks of toxicity versus stenting [179]. Additional studies comparing single-dose brachytherapy versus esophageal stenting had similar findings, with brachytherapy providing better long-term control of symptoms, fewer complications, and better quality of life scores versus stenting [180]. Dietary changes and endoscopic/ablative treatments may also relieve dysphagia related symptoms [177].

Although rare, complete esophageal obstruction (COE) can occur secondary to radiation treatment or due to unresectable tumor burden, limiting a patient’s ability to eat, drink, or swallow [181]. COE can cause severe pain, weight loss, aspiration pneumonia, and poor quality of life [182]. Various treatment approaches exist, with the immediate goal of restoring the esophageal lumen [183]. Esophageal reconstruction with combined antegrade-retrograde endoscopy is one such approach [184]. Placement of a J-tube or gastrostomy tube for nutrition and hydration is necessary if restoration of the esophageal lumen is unsuccessful [185,186]. Additional treatments for COE include EBRT, brachytherapy, and photodynamic therapy [187,188].

## 14. Cancer Surveillance

### 14.1. Surveillance Endoscopy

According to the NCCN and ACG, patients with superficial esophageal cancers (Tis, T1a, N0) status post (S/P) endoscopic resection or surgical resection should be surveyed via EGD every 3 months for the first year, every 6 months for the second year, and then annually for life [72,91]. Ablation therapy is indicated before surveillance for any patient with incompletely resected BE. Patients with T1b, N0 S/P endoscopic ablation should be surveyed via EGD every 3 months for the first year, every 4–6 months for the second year, and then annually for life [91].

For patient’s S/P surgical resection and adjuvant therapy for locally advanced EAC with residual BE, ablation therapy is recommended, followed by 3mo/6mo/annual indefinite surveillance EGD. Conversely, patients with locally advanced EAC S/P surgical resection and neoadjuvant CRT/CTX only require EGD as clinically indicated. Patients S/P dCRT without surgical resection should be surveyed via EGD every 3–6 months for 2 years and then annually for 3 additional years [91]. Relapse is common in patients treated with bimodal therapy (BMT), such as dCRT [189,190]. In one prospective cohort study of 276 patients undergoing BMT for EAC, 66.7% of patients experienced relapse, with 53% of the patients with initial negative surveillance EGD and PET/CT relapsing within the 5-year study period [191]. About 98% of relapses occurred within the first 3 years of surveillance.

### 14.2. Surveillance Imaging

Imaging studies are not standard for surveillance of superficial cancers (Tis, T1a, N0) [91,192,193]. For all other stages, the NCCN recommends surveillance imaging (CT chest/abdomen with oral and IV contrast) every 3–6 months for the initial 2 years and then annually for up to 5 years S/P treatment [190].

## 15. Discussion

Despite advancements in the understanding and treatment of esophageal adenocarcinoma over the last decade, the annual incidence continues to increase with little change to overall survival. Disease recurrence and progression after curative treatment are common. The high proportion of late-stage diagnoses and the poor success of surveillance programs highlight the limitations of current recommended protocols. Improving means of early detection is the first step in achieving better outcomes for patients with esophageal adenocarcinoma. Noninvasive, in-office screening tools (i.e., swallowable cell-collection devices or biomarker assays) warrant further investigation to evaluate their potential role in future screening protocols. Due to the significant variability in the expression of various biomarkers among patients, a comprehensive approach to risk stratification is required to predict progression from Barrett’s esophagus to esophageal adenocarcinoma and to allow for more personalized surveillance strategies. Aberrant expression of p53 protein, as determined by the immunostaining of the biopsy or resected tissue sections, is a promising biomarker for risk stratification. Hypermethylation of specific genes could also be used as a diagnostic and predictive tool for of Barrett’s progression and associated dysplasia.

The discovery of actionable biomarkers has transformed the current landscape of oncology and healthcare at large. Continual investigation into immunotherapeutic targets of malignancy is of great therapeutic and prognostic value. Overall, the genetic and epigenetic alterations in several genes and proteins during the progression of intestinal metaplasia to low-grade dysplasia and high-grade dysplasia to esophageal adenocarcinoma provide an opportunity to identify several biomarkers in the blood, mucus, and tissues based on the histochemical changes, genomic instability, proteomic and metabolomic studies. Accordingly, careful histological and proteomic analyses of resected lesions with the use of molecular diagnostics would be helpful in the development of treatment strategies.

Progress towards better outcomes requires a multimodal approach. Translational research, coupled with improved risk stratification, cost-effective screening protocols, and a multidisciplinary approach to patient care offers the best opportunity to meaningfully increase progression-free survival, reduce recurrence, and enhance quality of life for patients with esophageal adenocarcinoma.

## Figures and Tables

**Table 1:** American Joint Committee on Cancer (AJCC) Staging System, 8^th^ Edition^a^

T category	Depth of invasion of primary tumor
Tis	High-grade dysplasia, malignant cells confined to the epithelium by the basement membrane.
T1	Tumor invades the lamina propria (T1a), muscularis mucosa (T1a), or submucosa (T1b).
T2	Tumor invades the muscularis propria.
T3	Tumor invades the adventitia.
T4	Tumor invades adjacent structures (T4a: resectable; T4b: unresectable).
**N category**	**Lymph node involvement**
N0	No regional lymph node metastasis.
N1	1 to 2 positive regional lymph nodes.
N2	3 to 6 positive regional lymph nodes.
N3	7 or more positive regional lymph nodes.
**M category**	**Extent of metastatic disease**
M0	No distant metastasis.
M1	Distant metastasis.
**G category**	**Histological grade**
G1	Well differentiated.
G2	Moderately differentiated.
G3	Poorly differentiated.

a Data extracted from published report [89]

**Table 2:** Siewert Classification for Tumors of the Esophagogastric Junction^a^

Siewert Type	Description
**Siewert Type I**	Tumor epicenter located within 1 cm to 5 cm above the anatomic EGJ.
**Siewert Type II**	Tumor epicenter within 1 cm above and 2 cm below the EGJ.
**Siewert Type III**	Tumor epicenter between 2 cm and 5 cm below the EGJ, which infiltrates the EGJ and lower esophagus from below.

a Data extracted from published report [90]

## Key Points

- Barrett’s esophagus is the precancerous condition preceding esophageal adenocarcinoma, characterized by intestinal metaplasia of the distal esophagus. History of chronic gastroesophageal reflux disease is the best understood risk factor for developing Barrett’s esophagus, but nearly half of patients receiving a diagnosis present asymptomatically and without a history of chronic reflux.
- The accumulation of genetic mutations and epigenetic alterations drives the metaplasia-dysplasia-carcinoma sequence.
- Esophageal adenocarcinoma carries a 5-year survival rate of approximately 20%. Risk factors include chronic reflux, older age, male sex, obesity, and tobacco smoking.
- Approximately 90% of EAC diagnoses are made outside of structured surveillance programs, with nearly 40% of patients initially diagnosed at stage IV.
- Multimodal therapy is recommended for patients with locally advanced EAC, consisting of perioperative chemotherapy or neoadjuvant chemoradiotherapy, surgical resection, and in some cases adjuvant or neoadjuvant immunotherapy.
- The ESOPEC phase III trial demonstrated a significant overall survival advantage for treating locally advanced EAC with perioperative chemotherapy plus surgical resection compared to neoadjuvant chemoradiotherapy plus surgical resection.
- Various molecular-targeted therapies for adenocarcinoma of the esophagus or esophagogastric junction now exist, including PD1-blocking antibodies, CLDN18.2- directed cytolytic antibodies, VEGFR2 antagonists, monoclonal antibodies for HER2-positive tumors, and tyrosine kinase inhibitors for NTRK gene fusion.
- Improving mechanisms for early detection with risk-stratified, cost-effective screening tools is the first step in achieving better outcomes for patients with esophageal adenocarcinoma.
